# Advancing Cryo-EM and Cryo-ET through Innovation in Sample Carriers: A Perspective

**DOI:** 10.1021/acs.analchem.5c01534

**Published:** 2025-06-06

**Authors:** Navya Premaraj, Ron M. A. Heeren, Raimond B. G. Ravelli, Kèvin Knoops

**Affiliations:** † Maastricht MultiModal Molecular Imaging Institute(M4I), 82246 Maastricht University, Maastricht 6229 ER, The Netherlands; ‡ Microscopy CORE Lab, Maastricht University, Maastricht 6229 ER, The Netherlands

## Abstract

Cryo-electron microscopy (cryo-EM) and cryo-electron tomography (cryo-ET) have revolutionized structural biology by enabling high-resolution imaging of biomolecules and cellular structures. However, traditional sample carriers, such as copper grids with carbon films, presented limitations, particularly in cryo-ET workflows. Issues like uneven cell distribution, beam-induced motion, and suboptimal vitrification can compromise data quality. Recent advances in sample carrier design have successfully addressed key challenges, including the development of gold-based supports, graphene coatings, and nanofluidic chips. These innovations have improved mechanical stability, enhanced thermal conductivity, and provided better control over ice layer uniformity, leading to more consistent sample preparation and higher-quality imaging. In this perspective, sample preparation advancements, novel approaches such as titanium autogrids and slot grids with continuous gold foils, and their role toward future cryo-ET applications are discussed. These new designs have the potential to simplify workflows and optimize cell growth environments. Furthermore, this perspective highlights how integrating these cutting-edge technologies with prior advancements in sample carrier design can enhance cryo-ET workflows. Combined, they enable cryo-EM imaging of thicker samples and drive progress in structural biology research.

## Graphical abstract

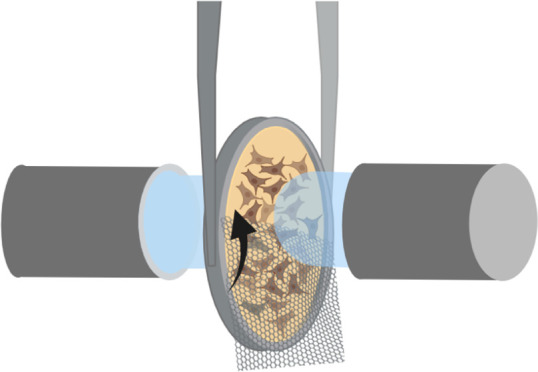

## Introduction

The ability to preserve biological specimens in their native, hydrated states through vitrification is fundamental to the success of cryo-electron microscopy (cryo-EM).^1^ This revolutionary imaging technique has enabled the study of macromolecules, cellular structures, and entire organisms at near-atomic resolution.^2–5^ Central to cryo-EM’s efficacy are sample carriers, grids designed to hold vitrified samples within the electron microscope. Since their introduction in the 1930s, cryo-EM sample carriers have undergone continuous refinement, evolving from basic copper nets^6^ to the standard, amorphous carbon foils suspended across a metal mesh grid. This development led to the first successful images of vitrified biological samples, setting the benchmark for cryo-EM specimen support.^7^ Over time, various modifications of support films have been introduced and utilized, further improving the performance and capabilities of cryo-EM in capturing high-resolution structural details. While these carriers facilitated groundbreaking discoveries in structural biology, their fundamental design has remained largely unchanged for decades and have not evolved to meet the increasingly demanding requirements of modern cryo-ET.

The preparation of cryo-EM specimens requires careful optimization of a variety of parameters, and achieving reproducible conditions is a challenging and time-consuming process. Even after finding the optimal conditions for vitrification, producing uniform thin layers of ice with properly distributed particles remains difficult. Issues such as preferential orientation, particle distortion at the air–water interface, and beam-induced motion in the SPA workflow have posed significant challenges.^8–14^ Efforts to address these problems have driven substantial advancements in substrate materials, sample carrier design, detectors, and software algorithms.^8,13,15–19^ However, the conventional grid structure still presents significant limitations for cryo-ET. Cryo-ET workflows demand rapid cooling to achieve effective vitrification. However, the thermal mass introduced by metal grid bars can hinder this process, a challenge that becomes more pronounced due to the increased thickness of samples in cryo-ET. Additionally, the grid bars obstruct high-angle data acquisition, which is crucial for obtaining 3D reconstructions in tomography. Moreover, when cells are grown on EM grids, they often adhere preferentially to the grid bars rather than the central support film, making much of the sample unsuitable for cryo-ET analysis.^20^ This issue poses a particular challenge in cryo-focused ion beam (cryo-FIB) milling, a technique used to prepare specimens for high-resolution imaging.^21^

Innovative solutions such as micropatterning have emerged to address these problems. Micropatterning involves surface modifications that guide cells to adhere selectively to the central support film while minimizing their attachment to the grid bars. Techniques like surface functionalization with biomolecules (e.g., fibronectin or laminin) on the support film, combined with antifouling treatments (e.g., polyethylene glycol (PEG)) on the grid bars, create differential adhesion environments.^22^ Other approaches, such as adjusting the surface hydrophilicity to make the central film more cell-attractive than the grid bars or adding topographical features like grooves or ridges, further enhance cell adherence to the desired areas.^20,22–26^ These modifications increase the proportion of usable samples, reduce artifacts, and improve consistency in sample preparation.

Despite these advancements, micropatterning introduces additional complexity and cost due to the advanced fabrication, coating, and treatment steps required. These additional processes can lengthen the preparation time and increase the overall expense of sample preparation. Consequently, traditional sample carriers continue to dominate, despite their limitations.

These sample preparation challenges have direct consequences for downstream data processing and resolution outcomes. Uneven cell distribution can lead to local variations in ice thickness and particle concentration, complicating particle picking and leading to biases in particle orientation. Beam-induced motion, particularly if non-uniform across the field of view, can degrade the signal-to-noise ratio and hinder the accuracy of alignment in both SPA and subtomogram averaging. Similarly, inconsistent vitrification can result in variable ice quality and compression artifacts, which can obscure structural details and introduce variability that reduces the resolution of averaged structures. Addressing these issues is therefore critical not only for sample preservation but also for achieving the high-resolution goals of modern cryo-EM studies.

There is a growing need for sample carriers specifically designed for cellular studies to overcome these challenges and streamline the preparation process. Researchers are increasingly exploring alternatives to standard mesh grids, particularly for protein samples through applications such as liquid-phase electron microscopy and MEMS chip.^27–30^ These approaches are opening up new possibilities for studying samples in the liquid phase and for imaging dynamic processes. Such innovations in cryo-ET sample carriers have the potential to transform the field by enhancing vitrification efficiency, improving sample positioning, and simplifying the overall process. This perspective will explore key innovations in sample carrier design from the broader field and highlights some specific research developments related to this topic. It also discusses the opportunities these innovations present for advancing cryo-ET research in the future.

### Innovations in Sample Carriers for Cryo-EM

Recent advancements in cryo-EM have led to innovative sample carriers that address challenges like beam-induced motion and the air–water interface.

### Advancements in Substrate Materials

Specimen movement during electron beam exposure has long been a significant obstacle, causing image blurring and the loss of high-resolution data. Advances such as direct electron detectors and motion correction algorithms have provided critical insights into and compensation for radiation-induced particle movement.^16,31^ However, the instability of conventional amorphous carbon substrates, which tend to bend and deform within the substrate plane, posed additional challenges.^8,32,33^

To address this issue, alternative materials have been explored. For example, titanium–silicon films have demonstrated a 50% reduction in movement, while doped silicon carbide has shown comparable improvements.^34–36^ Silicon dioxide (SiO_2_) is commonly used as a substrate in cryo-EM due to its excellent electron transparency, smooth surface, and biocompatibility.^37,38^

Graphene has emerged as a particularly promising substrate due to its exceptional mechanical strength, electron transparency, and atomic thinness.^39^ Initially, its inherent hydrophobicity hindered widespread application, but recent developments such as plasma treatment have enhanced protein adsorption, significantly improving image quality.^36^ Ultraflat graphene substrates now offer a stable and uniform platform for vitreous ice formation, where the amorphous ice and sample form a continuous film rather than a suspended puck of ice within a hole, as seen with traditional holey grids. This configuration enables high-resolution data collection for smaller proteins.^40^ It also prevents bulging and doming of ice in the hole under beam irradiation which can cause beam induced motion.^41^ Sample charging is a contributing factor to beam-induced motion, and the use of graphene-coated grids has been shown to significantly reduce this.^18^ Furthermore, depositing a conductive graphene layer and utilizing a smaller beam size effectively mitigates charging, leading to improved imaging stability.^42^ Recent developments in silicon-based carriers with a monolayer of graphene are gaining attention to combine MEMS technology with traditional grid formats. The rigid silicon frame improves handling and sample preparation, while microfabrication ensures an atomically flat, scalable, and reproducible surface. These carriers have shown significantly reduced beam-induced motion, especially in the early frames, thereby preserving data quality.^43^

These advancements highlight the increasing promise of graphene as a transformative substrate for cryo-EM applications. However, existing designs have not fully prevented substrate movement and remain difficult to manufacture and utilize. This led to further exploration of substrates capable of significantly minimizing radiation-induced deformation in thin, ice-embedded specimens at cryogenic temperatures. Such innovations effectively reduce both perpendicular and in-plane motion during imaging, thereby improving image quality for all radiation-sensitive cryogenic specimens. While graphene offers notable advantages, the challenges in its application have paved the way for alternative materials like gold, which address many of these limitations with unique properties.

### Ultra-Gold Supports

Typical Quantifoil supports suffer from significant movement upon irradiation, primarily due to crinkling of carbon foils caused by differential contraction between the carbon film and the metal grid during cooling.^44^ This results in inconsistent behavior across different grid holes. Additionally, amorphous carbon films, being semiconducting with highly variable resistivity, accumulate static and semi-mobile charges at cryogenic temperatures, exacerbating instability and image distortion.

Gold-based supports have provided a revolutionary solution by eliminating differential contraction using gold for both the foil and the mesh grid. Gold’s superior electrical conductivity prevents charge accumulation, while its excellent thermal conductivity ensures efficient heat dissipation. Additionally, irradiation generates secondary electrons that neutralize positive charges in the specimen.^36^ Furthermore, gold exhibits unmatched radiation hardness, maintaining structural integrity under intense electron bombardment, and its biocompatibility ensures minimal interference with biological samples.

These supports are made from a 3 mm gold disk as shown in Figure 1A, with a perforated polycrystalline gold foil and show dramatically reduced vertical motion under irradiation compared to amorphous carbon grids by 60-fold without ice (228 Å for am-C vs 3.8 Å for gold) and by 40-fold with ice (76 Å for am-C vs 1.9 Å for gold) under standard cryo-EM conditions.^19,36^ This enhanced stability and reduced distortion significantly improve image quality. Ongoing research aims to refine these designs to push the boundaries of high-resolution cryo-EM imaging even further. However, despite the impressive stability provided by gold supports, achieving uniformity in sample preparation, especially at the air–water interface, remained a significant challenge in cryo-EM workflows.

**Figure 1:**
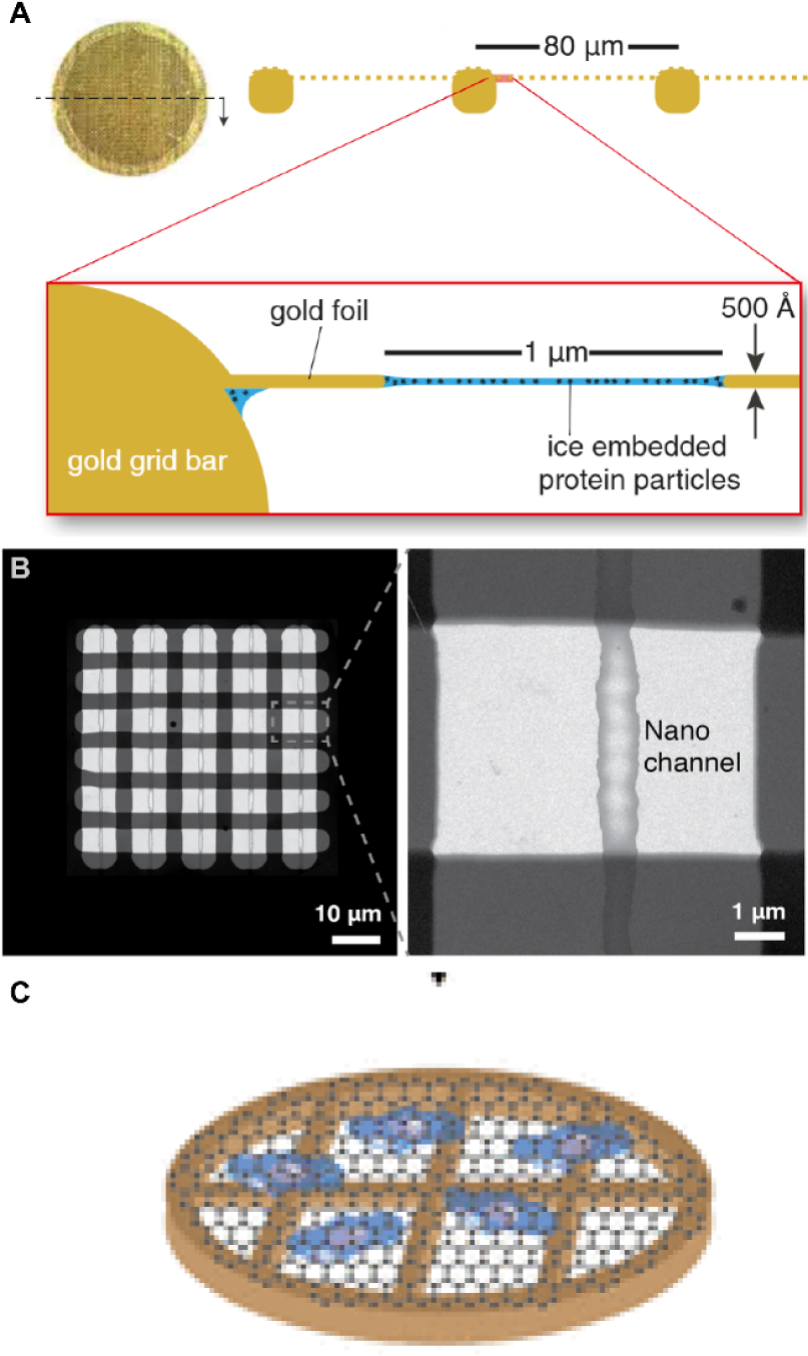
Advancements in sample carriers. (A) UltraAufoil grids, composed entirely of gold, featuring a 3 mm disc with a square mesh that supports a 500 Å thick foil with a regular array of micrometer-sized holes. Image reproduced from Russo et al., *J. Struct. Biol.*, **2016**, *193* (1), 33–44. Licensed under CC BY 4.0.^44^ (B) TEM image of the cryoChip observation membrane, showcasing five nanochannels designed for automated data acquisition. Inset: Close-up view of the nanochannels. Image reproduced from Huber et al., *eLife*, **2022**, *11*, e72629. Licensed under CC BY 4.0.^45^ (C) Illustration of local graphene liquid cells (GLCs) encapsulating the growth solution. Image reproduced with permission from reference,^46^ permission obtained via RightsLink.

### Addressing Uniformity and the Air–Water Interface with MEMS Nanofluidic Chips

Traditional methods like plunge freezing often lead to inconsistent results, mainly due to variations in blotting. Although alternatives, such as suction techniques,^47^ have been introduced, inconsistencies persist. Alternative approaches, including self-wicking grids, spray-based methods, and direct thin-film dispensing (e.g., pin printing, cryoWriters, or inkjet dispensers),^48–55^ have shown promise but remain constrained by the limitations of grid-based supports.

The development of liquid-phase transmission electron microscopy (LP-EM) introduced the ability to image liquids in electron microscopes by enclosing them within liquid cells.^29,56^ It showed that the challenges posed by the air–water interface and sample uniformity could be completely eliminated if the sample were enclosed within a membrane that is sufficiently electron-transparent, enabling high-contrast imaging of weak-phase objects. This would also simplify the sample application process, removing the need for blotting or wicking. LP-EM has already demonstrated its potential by being applied to a wide variety of systems, including biological specimens, significantly advancing the capability of electron microscopy to study liquid.^57–62^ However, thick silicon nitride (SiN) membranes in LP-EM cells limited resolution due to additional scattering. Inspired by the behavior of solidified water and LP-EM, nanofluidic sample cells with ultrathin SiN membranes have emerged as a promising alternative with the potential to automate cryo-EM workflow.^45^ MEMS-based nanofluidic chips now provide electron-transparent nanochannels that address the air–water interface issue, ensuring reproducible control over ice layer thickness (Figure 1B). These chips ensure uniform imaging conditions by standardizing the nanochannel width and height across individual chips, although they do not claim to achieve complete vitrification.^45^ Integrating this approach with jet vitrification has the potential to further improve vitrification quality, marking a significant advancement in single-particle cryo-EM sample preparation.

### Graphene Liquid Cells

Graphene liquid cells is another technique emerging as a transformative tool in cryo-EM. Leveraging graphene’s exceptional mechanical strength,^63^ atomic thinness, and superior electrical conductivity, entrapping samples in these cells overcome long-standing challenges in sample preparation.^39^ By enclosing minute volumes of liquid between two graphene layers, van der Waals forces trap the liquid within well-defined regions, enabling atomic-resolution imaging unattainable with traditional silicon nitride membranes. Conventional materials used for liquid cells, such as silicon nitride or silicon oxide, require thick layers that hinder electron transmittance. In 2012, graphene’s ability to encapsulate liquids was first demonstrated by studying the formation of platinum nanocrystals in graphene liquid cells at atomic resolution.^64^

Graphene’s electron transparency minimizes energy loss, preserving sample integrity during imaging. Its mechanical strength withstands high pressures, while its impermeability isolates liquid samples from the vacuum environment of the microscope.^30^ Moreover, graphene mitigates damage caused by reactive species, such as radicals formed during electron beam interactions with water, by binding these radicals to the graphene layer.^65^ Additionally, its thermal conductivity reduces image drift caused by electron charging, ensuring stable and precise imaging.^42^ New designs for preparing graphene cells are continuously being explored,^66^ and direct observation of wet biological samples has already been demonstrated.^46^ A recent study introduces a cryo-to-liquid CLEM workflow that enables real-time imaging of biological materials in aqueous environments.^67^ This technique allows for the observation of beam-sensitive biological processes and dynamic molecular reactions with high precision.

Introduced in 2012, graphene liquid cells have set a new benchmark for resolution and sample stability in TEM.^64^ However, sample preparation for these remains a significant bottleneck, as the process is currently not highly reproducible and results tend to be user dependent. As research progresses, innovations in this domain hold great promise for advancing the field of cryo-EM by enabling high-resolution imaging and offering more reliable sample preparation methods, potentially through automated tools such as the VitroTEM system.

### Advancing Cryo-ET with Innovative Sample Carriers

Despite its transformative potential in structural biology, cryo-ET sample preparation workflows have seen limited advancement. Traditional mesh-based support grids and their autogrids remain the cornerstone of sample preparation, but there is room to explore alternatives that could simplify and enhance the process. Drawing inspiration from the innovative concepts discussed above, we propose additional approaches that address current challenges in sample preparation. In this perspective, these ideas, supported by preliminary results are outlined, which highlight their promise for improving cryo-ET workflows.

### Titanium (Ti) Autogrids and C-Clips: A Biocompatible Alternative

Titanium (Ti) autogrids were introduced as a biocompatible alternative to traditional copper AutoGrids. While copper has been the standard material for electron microscopy grids, its cytotoxic properties limit its suitability for direct cell cultivation. Titanium overcomes this limitation, enabling cells to grow directly on grids that are preclipped into autogrids, thereby simplifying workflows and reducing contamination risks.

This approach eliminates the need for handling bare grids, a cumbersome and contamination-prone step in traditional workflows. Instead, grids can be clipped onto autogrids in a dry state before introducing or culturing cells. This modification also removes the necessity of clipping grids under liquid nitrogen, a time-consuming process that is prone to handling errors such as grid breakage and folding. High cooling efficiency during ethane jet vitrification ensures that preclipped Ti autogrids are vitrified effectively,^55^ further optimizing the overall cryo-ET workflow. Dedicated toolsets for the clipping process, ensuring precise handling and user-friendly operation in support of these innovations have been developed. Figure 2 compares clipped Ti autogrids with standard copper AutoGrids, highlighting titanium’s biocompatibility and ease of handling. Additionally, as shown in Figure 3 live–dead staining of macrophages grown on Ti autogrids confirms cell viability, validating their suitability for cryo-ET applications. Ti autogrids are fully compatible with existing QuantifoilⓇ grids used in cryo-ET. When paired with jet vitrification, the preclipped Ti autogrids efficiently manage the added thermal mass, ensuring robust vitrification. This integration offers a reliable, user-friendly solution that enhances reproducibility and simplifies the cryo-ET sample preparation process.

**Figure 2:**
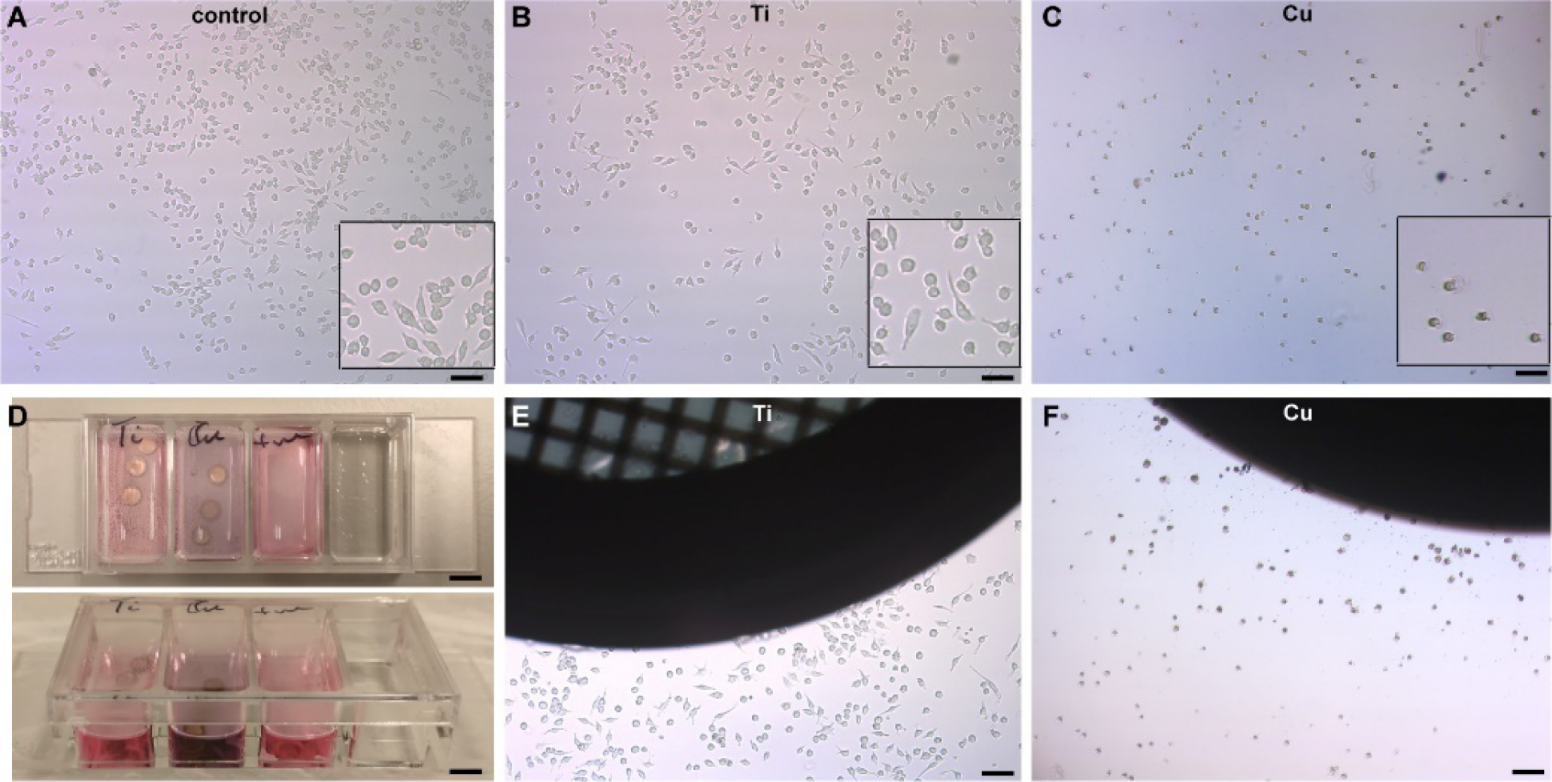
Ti autogrids mitigate biotoxicity. (A) Images of J774 macrophage cells grown in Petri dishes under control conditions, showing healthy cells. (B) Images of J774 macrophage cells grown in Petri dishes containing EM grids preclipped in Ti autogrids, where cells appear healthy and comparable to the control conditions, as observed in (E). (C) Images of J774 macrophage cells grown in Petri dishes containing EM grids preclipped in Cu AutoGrids, where cells exhibit significant differences compared to control conditions, with widespread cell death observed in the vicinity of the AutoGrid, as shown in (F). (D) Culture plate showing three columns: the first containing EM grids clipped in Ti autogrids, the second containing EM grids clipped in Cu AutoGrids, and the third as a control. The second column turns dark, correlating with the dead cells observed in (C) and (F). Scale bars: (A, B, C, E, F) is 50 μm, (D) is 5 μm.

**Figure 3:**
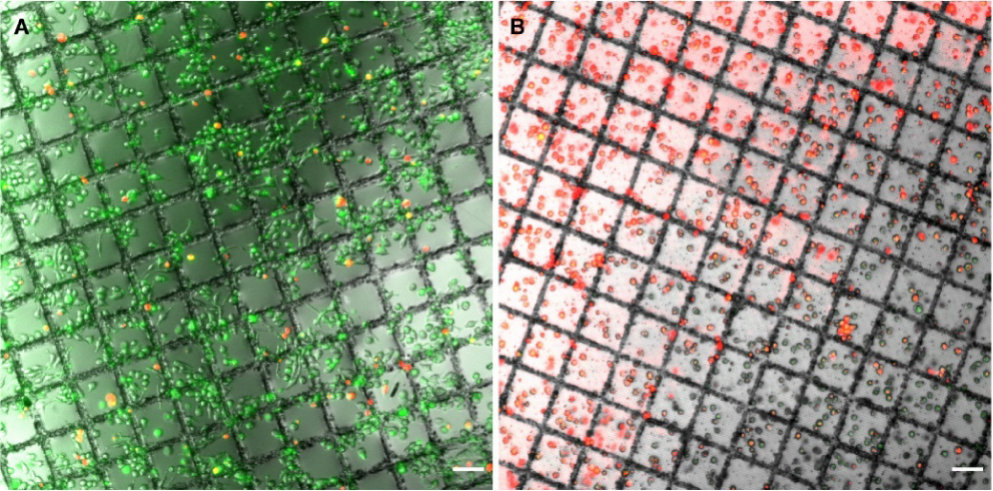
Live–dead staining of EM grids with cells grown on Ti and Cu autogrids. (A) Fluorescence images of J774 macrophage cells grown on UltraAuFoil R2/2 grids clipped with Ti autogrids, taken 3 h after seeding for adherence show predominantly green staining, indicative of healthy cells. (B) Images of J774 macrophage cells grown on UltraAuFoil R2/2 grids clipped with Cu AutoGrids, taken at the same time point, display significant red staining, indicative of cell death. Green fluorescence represents live cells, while red marks dead cells. Scale bar is 50 μm.

### Improved Sample Carriers for Cryo-ET: Slot Grids Overlaid with Au Foil Support Films and Continuous Au Foils

Advances in cryo-ET workflows can significantly benefit from optimized sample carriers that enhance cell growth, cooling efficiency, and sample integrity. Two promising approaches have been explored to address these challenges: slot grids with a 2 mm or 1 mm hole, covered with continuous gold (Au) foil support films, and continuous Au foils alone, preclipped onto titanium (Ti) autogrids. Both techniques aim to create flat, uniform surfaces for cell growth, (as shown in Figure 4) eliminate the interference of grid bars, and optimize thermal conductivity to facilitate rapid cooling. Together, these developments represent key steps toward advancing next-generation cryo-ET workflows by improving the preparation and preservation of samples.

**Figure 4:**
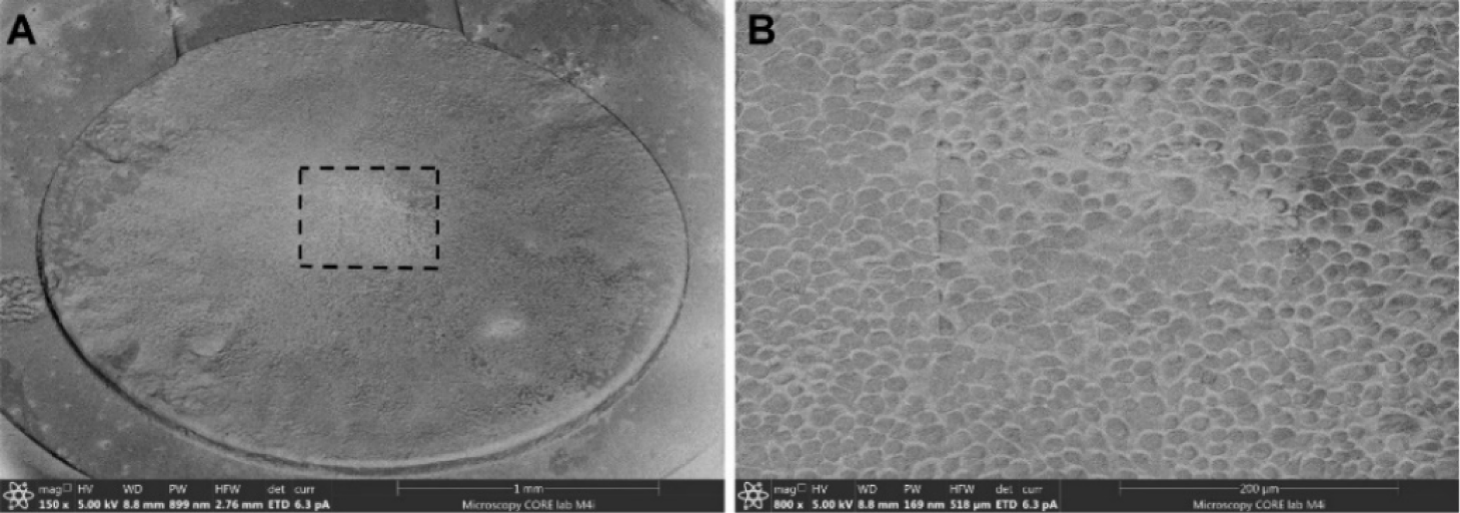
Continuous growth of cells on slot grids overlaid with Au foil. (A) Cryo-SEM image of the entire grid, showing the distribution of J774 macrophage cell lines across the surface. (B) Inset from the marked region in (A), showing a detailed view of the seamless and uninhibited distribution of cells on the Au foil.

The first method, slot grids overlaid with Au foil support films concentrates thermal mass at the edges of the grid. This contrasts with the irregular mass distribution of mesh grids, and helps to promote a more effective cooling gradient at the critical vitrification zone in the center.

Furthermore, the integration of jet vitrification optimizes cooling by creating a seamless gradient from the center outward. A novel workflow has been designed to prevent the backside of the grid from becoming wet during sample preparation. In this method, a concentrated drop of cell suspension is placed on a Petri dish, and the grids, with the Au foil side facing down, are placed on top of the drop for about an hour to facilitate adhesion. After this period, the grids are partially blotted, clipped, and a small amount of medium is applied to the cell side to avoid dehydration. The medium is then blotted from the front side just before vitrification. Figure 5 demonstrates the formation of lamellae from these grids, with vitrification quality validated using electron diffraction.

**Figure 5:**
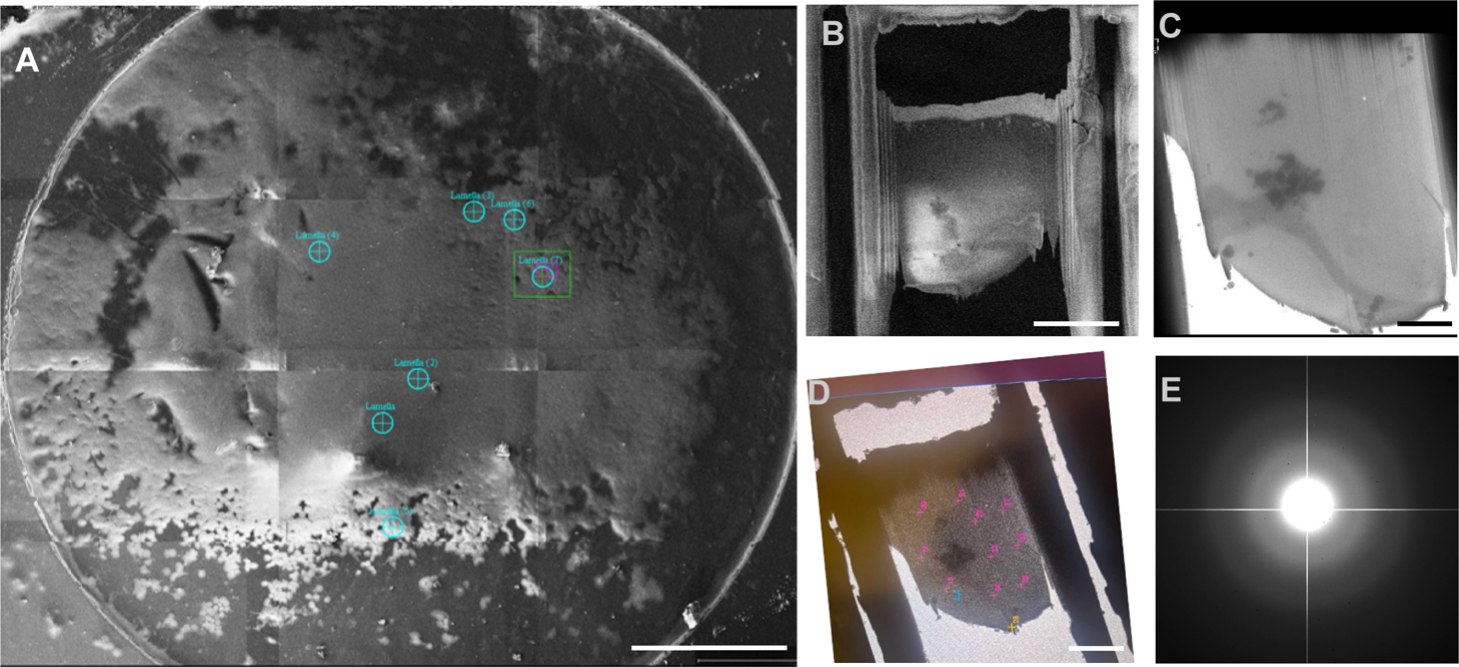
Cells grown on slot grids overlaid with continuous Au foil support films. (A) Cryo-SEM image of the entire clipped grid, with milling sites indicated. Images of milled lamellae acquired using (B) a Scios DualBeam FIB-SEM microscope and (C, D) a 200 kV Tecnai Arctica microscope at different magnifications. Part (D) shows areas marked for electron diffraction using the TimePix 3 detector. Diffraction patterns obtained from the marked areas confirmed the presence of amorphous ice, with one example shown in (E). The diffraction length was 360 mm, and the maximum resolution achieved was 1.31 Å. The scale bars are as follows: A – 500 nm, B and D – 5 μm, and C – 2 μm.

The second method involves using continuous gold (Au) foils alone, without slot grids, to eliminate the thermal mass of the underlying slot grid. However, this approach presents handling challenges, as the gold foils are too thin to be managed independently. Preclipping the foils onto titanium (Ti) autogrids offers a practical solution. Once preclipped, cells can be cultured or applied directly onto the Au foils, blotted, and subsequently subjected to jet vitrification techniques, with titanium being compatible, as previously mentioned. Since the samples are thin and lack the support of a grid bar, precise control of the two jets is crucial to prevent the sample from being blown away during the jet vitrification process. In earlier experiments, the thin foils were observed to be blown away in most cases. However, by enhancing the synchrony of the ethane jet, improved sample preparation was achieved.^68^ However, the inability to use Ti-autogrids in standard cryo-EM microscopes slowed the progress of these experiments, and consequently, data from these samples were not included.

Preliminary tests with Au foils of varying thicknesses; 400 nm, 1 μm, and 2 μm, highlight the advantages and challenges associated with each. The thinner 400 nm foils exhibited advantages in milling, producing fewer overhangs postmilling and achieving consistent vitrification across multiple points on the lamellae (see Figure 5). However, they posed challenges in maintaining flatness and were prone to folding. In contrast, the thicker 1 and 2 μm foils offered better mechanical stability but required longer milling times, particularly with gallium ion beams, and resulted in significant gold overhangs. Plasma-based milling systems, such as the Arctis-Cryo-Plasma FIB microscope, can significantly reduce milling time and improve the milling process.^69^ Furthermore, the ability to rotate sample holders using the 180° tilt capability of modern stages allows for rough milling from the backside, with the Au foil acting as a protective shield. This, combined with fluorescence imaging for prescreening regions of interest, can streamline workflows and improve milling precision.

The innovative sample carriers discussed above significantly streamline the sample preparation process for cryo-electron tomography (cryo-ET). Ti-autogrids eliminate the need to handle bare EM grids from the initial cell culture step and also remove the requirement for delicate post-vitrification handling, which typically demands significant expertise to avoid grid breakage or folding. The prepared grids appear intact, without any folds or cracks, as shown in Figure 3. Although the field of cryo-ET is rapidly evolving with the advent of integrative microscopes, it often remains a correlative technique involving the transfer of samples between different imaging platforms. In such workflows, starting with a stable and robust sample carrier is critical to minimizing the risk of damaging valuable specimens, especially those that have been grown, cultivated, or infected under specific expression protocols.

Furthermore, the use of slot grids with continuous gold foils provides a uniform surface for cell culture, free from mesh interruptions. This uniformity is particularly advantageous as the field moves toward more complex biological models, such as organoids,^70^ where maximizing usable sample area is essential. Compared to traditional mesh grids, these continuous substrates can support sample growth and offer more usable areas for cryo-correlative light and electron microscopy (cryo-CLEM) and complementary imaging techniques such as volume electron microscopy. Another key advantage of these improved sample carriers is their ability to reduce and evenly distribute thermal mass, enhancing heat dissipation in a more uniform manner. When combined with the high cooling efficiency of jet vitrification, this uniform cooling can support vitrification of even thicker samples. This approach could ultimately lead to a more robust, routine, and user-friendly sample preparation alternative for specimens in the 10–50 μm thickness range, avoiding the later complexities of the high-pressure freezing method and facilitating the widespread adoption of *in situ* structural biology.

### Integrating Graphene Technology for Enhanced Performance

Building on these improvements, integrating graphene technology presents a promising direction for addressing some of the mechanical limitations of Au-based carriers. Graphene, known for its exceptional electron transparency, mechanical strength, and sealing capabilities, offers tremendous potential in cryo-EM applications. Coating thin Au foils with graphene could enhance their structural integrity, potentially enabling the use of thinner foils, which improve heat transfer and cooling efficiency during vitrification, a critical step in cryo-EM workflows. Thinner foils also reduce milling times, further optimizing the sample preparation process.

Innovations such as graphene pockets, formed by layering graphene onto or enclosing samples on continuous gold foils, could serve as a complementary or alternative method. If layering graphene on top of or enclosing the sample helps retain minimal hydration after blotting, these pockets could streamline the optimization of blotting techniques, preserve sample hydration more effectively, and minimize the need for extensive fine-tuning of blotting parameters, a typically time-intensive step in cryo-EM sample preparation. While graphene’s low electron scattering may not directly impact cryo-ET imaging, as samples are milled prior to imaging, its remarkable mechanical strength and sealing properties make it an invaluable addition to cryo-EM workflows. Additionally, investigating other metals for continuous foils, such as titanium (Ti), presents promising advantages. Titanium, when used as a support material, has demonstrated its ability to provide rigid support, effectively withstanding the various stages of micropatterning, thereby improving stability and performance during sample preparation.^20^

These innovations in sample carriers for cryo-ET represent critical advancements in addressing longstanding challenges related to sample preparation, cooling, and milling. By optimizing support films, surface uniformity, and milling techniques, these methods not only enhance vitrification and sample integrity but also improve the overall efficiency and precision of cryo-ET workflows.

### Potential of Graphene Liquid Cells in Other Imaging Techniques

Graphene liquid cells (GLCs), which are being increasingly investigated as sample carriers in cryo-EM, also show great promise for enhancing other imaging techniques, such as scanning ion microscopy (SIMS). SIMS, which involves bombarding a sample surface with primary ions to eject secondary ions for mass spectrometry analysis, offers high-resolution chemical and elemental mapping of samples. GLCs, by maintaining biological samples in their native, hydrated state, create a stable environment for imaging, allowing for both structural and chemical data acquisition from the same sample. Recent studies have shown that GLCs enable SIMS imaging of untreated wet cell membranes, revealing molecular distributions such as cholesterol, phospholipids, and fatty acids at subcellular resolution, without the need for labeling.^71^ These findings demonstrate that GLCs can preserve cell integrity during analysis in ultrahigh-vacuum conditions, enabling the study of live cells and their molecular dynamics. As a result, GLCs are poised to expand the applicability of SIMS, offering new insights into the molecular composition and interactions within biological systems.

## Conclusion and Outlook

This perspective explores the continuous advancements in cryo-EM, focusing on the evolving role of sample carriers. While the primary focus has been placed on improving vitrification quality through jet vitrification, the complementary role of sample carriers remains critical. This has prompted an exploration of ways to develop carriers that support continuous cell growth or encapsulate thicker samples, aligning with the enhanced vitrification quality achieved through jet vitrification.

For instance, past jet freezing experiments validated the concept of sandwiching samples between metal foils to enhance vitrification.^72^ Revisiting these techniques in the context of modern advancements could unlock new possibilities for optimizing sample preparation. Key questions about the role of these sandwiching materials, such as whether their thermal conductivity accelerates cooling or their thermal mass hinders it, remain pivotal for further optimizing the vitrification strategy.

The evolution of sample carriers has not only underscored their significant impact on cryo-EM workflows but also highlighted opportunities for further investigation into their potential. The sample carrier designs discussed here lay the groundwork for advancing this exploration, particularly into the interplay between thermal properties and vitrification efficiency.

Moreover, concepts such as continuous gold foils and the integration of graphene into sample carrier designs present exciting opportunities to create hybrid systems. These systems could harness gold’s excellent thermal conductivity alongside graphene’s mechanical strength and minimal electron scattering, resulting in more efficient, robust, and versatile sample carriers. Such advancements hold significant potential for enhancing cryo-EM workflows and expanding their range of applications, paving the way for future innovations by building on earlier techniques.

When coupled with developments in vitrification techniques, fluorescence microscopy, and focused ion beam (FIB) milling technologies, these innovations could profoundly impact cryo-ET workflows. As the field increasingly seeks innovative alternatives, the importance of refining sample carriers cannot be overstated. This research offers the prospect of transforming cryo-ET workflows, making them more efficient, reproducible, and accessible for much thicker samples, thereby broadening their scope of applications. By fostering interdisciplinary collaborations across materials science, engineering, and structural biology, the field can fully realize the potential of these advancements, propelling cryo-EM to unprecedented heights.

## §

R.B.G.R. deceased on 30 June 2023.

## References

[1] Dubochet J., McDowall A. W. (1981). Vitrification of pure water for electron microscopy. J. Microsc..

[2] Bai X.-C., McMullan G., Scheres S. H. W. (2015). How
cryo-EM is revolutionizing structural
biology. Trends Biochem. Sci..

[3] Cheng Y. (2015). Single-Particle
Cryo-EM at Crystallographic Resolution. Cell.

[4] Renaud J.-P., Chari A., Ciferri C., Liu W.-T., Rémigy H.-W., Stark H., Wiesmann C. (2018). Cryo-EM in drug discovery: achievements,
limitations and prospects. Nat. Rev. Drug Discovery.

[5] Yip K. M., Fischer N., Paknia E., Chari A., Stark H. (2020). Atomic-resolution
protein structure determination by cryo-EM. Nature.

[6] Marton L. (1934). Electron Microscopy
of Biological Objects. Nature.

[7] Adrian M., Dubochet J., Lepault J., McDowall A. W. (1984). Cryo-electron microscopy
of viruses. Nature.

[8] Brilot A. F., Chen J. Z., Cheng A., Pan J., Harrison S. C., Potter C. S., Carragher B., Henderson R., Grigorieff N. (2012). Beam-induced motion of vitrified
specimen on holey
carbon film. J. Struct. Biol..

[9] Henderson R., Glaeser R. M. (1985). Quantitative analysis of image contrast in electron
micrographs of beam-sensitive crystals. Ultramicroscopy.

[10] Henderson R. (1995). The potential
and limitations of neutrons, electrons and X-rays for atomic resolution
microscopy of unstained biological molecules. Q. Rev. Biophys..

[11] Henderson R., Chen S., Chen J. Z., Grigorieff N., Passmore L. A., Ciccarelli L., Rubinstein J. L., Crowther R. A., Stewart P. L., Rosenthal P. B. (2011). Tilt-Pair
Analysis of Images from a Range of Different Specimens in Single-Particle
Electron Cryomicroscopy. J. Mol. Biol..

[12] Liu N., Wang H.-W. (2023). Better
Cryo-EM Specimen Preparation: How to Deal with
the Air–Water Interface?. J. Mol. Biol..

[13] Glaeser R. M., Hall R. J. (2011). Reaching the Information
Limit in Cryo-EM of Biological
Macromolecules: Experimental Aspects. Biophys.
J..

[14] Downing K. H., Glaeser R. M. (1986). Improvement in high resolution image
quality of radiation-sensitive
specimens achieved with reduced spot size of the electron beam. Ultramicroscopy.

[15] Glaeser R. M., McMullan G., Faruqi A. R., Henderson R. (2011). Images of
paraffin monolayer crystals with perfect contrast: Minimization of
beam-induced specimen motion. Ultramicroscopy.

[16] Li X., Mooney P., Zheng S., Booth C. R., Braunfeld M. B., Gubbens S., Agard D. A., Cheng Y. (2013). Electron counting and
beam-induced motion correction enable near-atomic-resolution single-particle
cryo-EM. Nat. Methods.

[17] Campbell M. G., Cheng A., Brilot A. F., Moeller A., Lyumkis D., Veesler D., Pan J., Harrison S. C., Potter C. S., Carragher B., Grigorieff N. (2012). Movies of Ice-Embedded Particles
Enhance Resolution in Electron Cryo-Microscopy. Structure.

[18] Böttcher B. (1995). Electron cryo-microscopy
of graphite in amorphous ice. Ultramicroscopy.

[19] Russo C. J., Passmore L. A. (2014). Ultrastable gold substrates for electron cryomicroscopy. Science.

[20] Toro-Nahuelpan M., Zagoriy I., Senger F., Blanchoin L., Théry M., Mahamid J. (2020). Tailoring cryo-electron
microscopy
grids by photo-micropatterning for in-cell structural studies. Nat. Methods.

[21] Rigort A., Bäuerlein F. J.
B., Villa E., Eibauer M., Laugks T., Baumeister W., Plitzko J. M. (2012). Focused ion beam
micromachining of eukaryotic cells for cryoelectron tomography. Proc. Natl. Acad. Sci. U. S. A..

[22] Sibert B. S., Kim J. Y., Yang J. E., Wright E. R. (2021). Micropatterning
Transmission Electron Microscopy Grids to Direct Cell Positioning
within Whole-Cell Cryo-Electron Tomography Workflows. J. Vis. Exp. JoVE.

[23] Damodaran V. B., Murthy N. S. (2016). Bio-inspired strategies for designing antifouling biomaterials. Biomater. Res..

[24] Sibert, B. S. ; Kim, J. Y. ; Yang, J. E. ; Wright, E. R. Whole-cell cryo-electron tomography of cultured and primary eukaryotic cells on micropatterned TEM grids. bioRxiv, 2021. 10.1101/2021.06.06.447251

[25] Swistak, L. ; Sartori-Rupp, A. ; Vos, M. ; Enninga, J. Chapter 3 - Micropatterning of cells on EM grids for efficient cryo-correlative light electron microscopy. In Methods in Microbiology; Gurtler, V. , Eds.; Academic Press, 2021, Vol. 48, pp. 95–110.

[26] Engel L., Gaietta G., Dow L. P., Swift M. F., Pardon G., Volkmann N., Weis W. I., Hanein D., Pruitt B. L. (2019). Extracellular
matrix micropatterning technology for whole cell cryogenic electron
microscopy studies. J. Micromech. Microeng..

[27] de
Jonge N., Ross F. M. (2011). Electron microscopy of specimens
in liquid. Nat. Nanotechnol..

[28] Ni M., Tong W. H., Choudhury D., Rahim N. A. A., Iliescu C., Yu H. (2009). Cell Culture on MEMS
Platforms: A Review. Int.
J. Mol. Sci..

[29] Peckys D. B., Macías-Sánchez E., de Jonge N. (2020). Liquid phase electron
microscopy of biological specimens. MRS Bull..

[30] Park J., Koo K., Noh N., Chang J. H., Cheong J. Y., Dae K. S., Park J. S., Ji S., Kim I.-D., Yuk J. M. (2021). Graphene
Liquid Cell Electron Microscopy: Progress, Applications, and Perspectives. ACS Nano.

[31] Bai X.-C., Fernandez I. S., McMullan G., Scheres S. H. W. (2013). Ribosome structures
to near-atomic resolution from thirty thousand cryo-EM particles. eLife.

[32] Wright E. R., Iancu C. V., Tivol W. F., Jensen G. J. (2006). Observations on
the behavior of vitreous ice at ∼ 82 and ∼ 12K. J. Struct. Biol..

[33] Booy F. P., Pawley J. B. (1993). Cryo-crinkling:
what happens to carbon films on copper
grids at low temperature. Ultramicroscopy.

[34] Rhinow D., Kühlbrandt W. (2008). Electron cryo-microscopy of biological specimens on
conductive titanium–silicon metal glass films. Ultramicroscopy.

[35] Yoshioka C., Carragher B., Potter C. S. (2010). Cryomesh: A New Substrate for Cryo-Electron
Microscopy. Microsc. Microanal..

[36] Russo C. J., Passmore L. A. (2014). Controlling protein
adsorption on graphene for cryo-EM
using low-energy hydrogen plasmas. Nat. Methods.

[37] Jasnin M., Ecke M., Baumeister W., Gerisch G. (2016). Actin Organization
in Cells Responding to a Perforated Surface, Revealed by Live Imaging
and Cryo-Electron Tomography. Structure.

[38] Maimon T., Elad N., Dahan I., Medalia O. (2012). The Human Nuclear Pore
Complex as Revealed by Cryo-Electron Tomography. Structure.

[39] Geim A. K. (2009). Graphene:
Status and Prospects. Science.

[40] Zheng L., Liu N., Gao X., Zhu W., Liu K., Wu C., Yan R., Zhang J., Gao X., Yao Y. (2023). Uniform
thin ice on ultraflat graphene for high-resolution cryo-EM. Nat. Methods.

[41] Naydenova K., Jia P., Russo C. J. (2020). Cryo-EM with sub–1
Å specimen movement. Science.

[42] Zhang Y., van Schayck J. P., Pedrazo-Tardajos A., Claes N., Noteborn W. E. M., Lu P.-H., Duimel H., Dunin-Borkowski R. E., Bals S., Peters P. J., Ravelli R. B. G. (2023). Charging of Vitreous
Samples in Cryogenic Electron Microscopy Mitigated by Graphene. ACS Nano.

[43] Papadimitriou V. A., Pechnikova E., Pen M., Perez Garza H. H. (2025). BPS2025
- Silicon-based cryo-EM grids: Revolutionizing sample stability and
data quality with microfabrication and graphene. Biophys. J..

[44] Russo C. J., Passmore L. A. (2016). Ultrastable gold substrates: Properties of a support
for high-resolution electron cryomicroscopy of biological specimens. J. Struct. Biol..

[45] Huber S. T., Sarajlic E., Huijink R., Weis F., Evers W. H., Jakobi A. J. (2022). Nanofluidic chips
for cryo-EM structure determination
from picoliter sample volumes. eLife.

[46] Park J., Park H., Ercius P., Pegoraro A. F., Xu C., Kim J. W., Han S. H., Weitz D. A. (2015). Direct Observation
of Wet Biological Samples by Graphene Liquid Cell Transmission Electron
Microscopy. Nano Lett..

[47] Koning R. A.-O., Vader H., van Nugteren M. A.-O.
X., Grocutt P. A., Yang W., Renault L. A.-O., Koster A. A.-O., Kamp A. C. F., Schwertner M. A.-O. (2022). Automated vitrification of cryo-EM
samples with controllable sample thickness using suction and real-time
optical inspection. Nat. Commun..

[48] Arnold S. A., Albiez S., Bieri A., Syntychaki A., Adaixo R., McLeod R. A., Goldie K. N., Stahlberg H., Braun T. (2017). Blotting-free and lossless cryo-electron microscopy grid preparation
from nanoliter-sized protein samples and single-cell extracts. J. Struct. Biol..

[49] Jain T., Sheehan P., Crum J., Carragher B., Potter C. S. (2012). Spotiton: A prototype for an integrated
inkjet dispense
and vitrification system for cryo-TEM. J. Struct.
Biol..

[50] Rima L., Zimmermann M., Fränkl A., Clairfeuille T., Lauer M., Engel A., Engel H.-A., Braun T. (2022). cryoWriter:
a blotting free cryo-EM preparation system with a climate jet and
cover-slip injector. Faraday Discuss.

[51] Rubinstein J. L., Guo H., Ripstein Z. A., Haydaroglu A., Au A., Yip C. M., Trani J. M. D., Benlekbir S., Kwok T. (2019). Shake-it-off: a simple
ultrasonic cryo-EM specimen-preparation device. Acta Crystallogr. D Struct. Biol..

[52] Wei H., Dandey V. P., Zhang Z., Raczkowski A., Rice W. J., Carragher B., Potter C. S. (2018). Optimizing “self-wicking”
nanowire grids. J. Struct. Biol..

[53] Feng X., Fu Z., Kaledhonkar S., Jia Y., Shah B., Jin A., Liu Z., Sun M., Chen B., Grassucci R. A. (2017). A Fast and Effective Microfluidic Spraying-Plunging Method for High-Resolution
Single-Particle Cryo-EM. Structure.

[54] Kontziampasis D., Klebl D. P., Iadanza M. G., Scarff C. A., Kopf F., Sobott F., Monteiro D. C. F., Trebbin M., Muench S. P., White H. D. (2019). A cryo-EM grid preparation
device for time-resolved
structural studies. IUCrJ.

[55] Ravelli R. B. G., Nijpels F. J. T., Henderikx R. J. M., Weissenberger G., Thewessem S., Gijsbers A., Beulen B. W. A. M. M., López-Iglesias C., Peters P. J. (2020). Cryo-EM structures from sub-nl volumes using pin-printing
and jet vitrification. Nat. Commun..

[56] Peckys D. B., Veith G. M., Joy D. C., Jonge N. D. (2009). Nanoscale Imaging
of Whole Cells Using a Liquid Enclosure and a Scanning Transmission
Electron Microscope. PLoS One.

[57] Jonge N. D., Peckys D. B., Kremers G. J., Piston D. W. (2009). Electron microscopy
of whole cells in liquid with nanometer resolution. Proc. Natl. Acad. Sci. U. S. A..

[58] Nishiyama H., Suga M., Ogura T., Maruyama Y., Koizumi M., Mio K., Kitamura S., Sato C. (2010). Atmospheric scanning electron microscope
observes cells and tissues in open medium through silicon nitride
film. J. Struct. Biol..

[59] Mirsaidov U. M., Zheng H., Casana Y., Matsudaira P. (2012). Imaging Protein
Structure in Water at 2.7 nm Resolution by Transmission Electron Microscopy. Biophys. J..

[60] Varano A. C., Rahimi A., Dukes M. J., Poelzing S., McDonald S. M., Kelly D. F. (2015). Visualizing virus particle mobility
in liquid at the
nanoscale. Chem. Commun..

[61] Wang X., Yang J., Andrei C. M., Soleymani L., Grandfield K. (2018). Biomineralization of calcium phosphate revealed by
in situ liquid-phase electron microscopy. Commun.
Chem..

[62] De
Yoreo J. J. (2016). In-situ liquid phase TEM observations of nucleation
and growth processes. Prog. Cryst. Growth Charact.
Mater..

[63] Lee C., Wei X., Kysar J. W., Hone J. (2008). Measurement of the Elastic Properties
and Intrinsic Strength of Monolayer Graphene. Science.

[64] Yuk J. M., Park J., Ercius P., Kim K., Hellebusch D. J., Crommie M. F., Lee J. Y., Zettl A., Alivisatos A. P. (2012). High-Resolution
EM of Colloidal Nanocrystal Growth Using Graphene Liquid Cells. Science.

[65] Cho H., Jones M. R., Nguyen S. C., Hauwiller M. R., Zettl A., Alivisatos A. P. (2017). The Use of Graphene and Its Derivatives
for Liquid-Phase Transmission Electron Microscopy of Radiation-Sensitive
Specimens. Nano Lett..

[66] Kelly D. J., Zhou M., Clark N., Hamer M. J., Lewis E. A., Rakowski A. M., Haigh S. J., Gorbachev R. V. (2018). Nanometer
Resolution Elemental Mapping in Graphene-Based TEM Liquid Cells. Nano Lett..

[67] Rutten L., Joosten B., Schaart J., de Beer M., Roverts R., Gräber S., Jahnen-Dechent W., Akiva A., Macías-Sánchez E., Sommerdijk N. (2025). A Cryo-to-Liquid Phase Correlative Light Electron Microscopy
Workflow for the Visualization of Biological Processes in Graphene
Liquid Cells. Adv. Funct. Mater..

[68] Premaraj N., Huysmans P., Heeren R. M. A., Ravelli R. B. G., Knoops K. (2025). Dual-jet synchronization
results in higher cooling rates for sample vitrification. Phys. Fluids.

[69] Berger C., Watson H., Naismith J. H., Dumoux M., Grange M. (2025). Xenon plasma
focused ion beam lamella fabrication on high-pressure frozen specimens
for structural cell biology. Nat. Commun..

[70] Hoffmann P. C., Giandomenico S. L., Ganeva I., Wozny M. R., Sutcliffe M., Lancaster M. A., Kukulski W. (2021). Electron cryo-tomography reveals
the subcellular architecture of growing axons in human brain organoids. eLife.

[71] Lim H., Lee S. Y., Park Y. A.-O., Jin H., Seo D. A.-O., Jang Y. H., Moon D. A.-O. (2021). Mass spectrometry imaging of untreated
wet cell membranes in solution using single-layer graphene. Nat. Methods.

[72] Burstein N. L., Maurice D. M. (1978). Cryofixation of tissue surfaces by
a propane jet for
electron microscopy. Micron (1969).

